# Accumulation of Mitochondrial *RPPH1* RNA Is Associated with Cellular Senescence

**DOI:** 10.3390/ijms22020782

**Published:** 2021-01-14

**Authors:** Ji Won Lee, Yoo Lim Chun, Ah Young Kim, Lawson T. Lloyd, Seungbeom Ko, Je-Hyun Yoon, Kyung-Won Min

**Affiliations:** 1 Department of Biology, College of Natural Sciences, Gangneung-Wonju National University, Gangneung 25457, Korea; wldnjs1502@gmail.com (J.W.L.); kayp8@hanmail.net (A.Y.K.); 2 Department of Biochemistry and Molecular Biology, Medical University of South Carolina, Charleston, SC 29425, USA; narumi127@naver.com (Y.L.C.); lawsontlloyd@gmail.com (L.T.L.); bleddu@naver.com (S.K.); yoonje@musc.edu (J.-H.Y.); 3 Department of Anatomy and Neurobiology, College of Medicine, Kyung Hee University, 26, Kyungheedae-ro, Dongdaemun-gu, Seoul 02447, Korea

**Keywords:** mitochondrial long noncoding RNA, RNA-binding protein, posttranscriptional gene regulation, cellular senescence

## Abstract

Post-transcriptional gene regulation is an important step in the regulation of eukaryotic gene expression. Subcellular compartmentalization of RNA species plays a crucial role in the control of mRNA turnover, spatial restriction of protein synthesis, and the formation of macromolecular complexes. Although long noncoding RNAs (lncRNAs) are one of the key regulators of post-transcriptional gene expression, it is not heavily studied whether localization of lncRNAs in subcellular organelles has functional consequences. Here, we report on mitochondrial lncRNAs whose expression fluctuates in the process of cellular senescence. One of the mitochondrial lncRNAs, *RPPH1* RNA, is overexpressed and accumulates in mitochondria of senescent fibroblasts, possibly modulated by the RNA-binding protein AUF1. In addition, *RPPH1* RNA overexpression promotes spontaneous replicative cellular senescence in proliferating fibroblasts. Using MS2 aptamer-based RNA affinity purification strategy, we identified putative target mRNAs of *RPPH1* RNA and revealed that partial complementarity of *RPPH1* RNA to its target mRNAs prevents those mRNAs decay in proliferating fibroblasts. Altogether, our results demonstrate the role of mitochondrial noncoding RNA in the regulation of mRNA stability and cellular senescence.

## 1. Introduction

Eukaryotic gene expression is tightly regulated in transcription and post-transcriptional steps [1,2]. Post-transcriptional gene regulation is mainly governed by RNA-binding proteins (RBPs) and noncoding RNAs (ncRNAs) in pre-mRNA splicing, export from nucleus, localization in specific cellular sites, RNA decay, modification, and translation [3,4]. RBPs recognize specific sequences in open-reading frames and untranslated regions to determine the fate of target RNAs [5]. Recent advances in RNA sequencing technology enabled identification of target RNA sequences protected by RBPs with single-nucleotide resolution [6]. In addition to small ncRNAs (e.g., small interfering RNAs, microRNAs, piwi-interacting RNAs, etc.), but lncRNAs with size greater than 200 nt also utilize their unique sequences to recognize target mRNAs for degradation or repression in translation [7,8,9]. Modification of cross-linking immunoprecipitation (CLIP) method also allowed the determination of exact target RNA sequences by utilizing ligation of ncRNAs and mRNAs interacting with RBPs [10,11]. Altogether, the mechanism of post-transcriptional gene regulation is heavily studied through advances in high throughput RNA sequencing technology.

Even though amounts of total transcripts in cells may determine efficiency of protein output, additional factors also contribute critically in protein production [12]. For example, mRNA decay rates influence availability of transcripts recognized by the ribosome, and translation rates also affect mRNA translation efficiency in the quantity of a given mRNA transcripts [13,14]. More importantly, localization of mRNAs governs the accessibility of mRNAs for binding to translation initiation factors and 43S pre-initiation complex [15,16]. Cytoplasmic mRNA granules such as P-bodies and stress granules are proposed to function in regulation of mRNA decay and translation through increasing local concentration of mRNAs and proteins [17,18]. mRNAs also can localize in cellular organelles such as mitochondria and endoplasmic reticulum for more restricted availability for their stability and translation regulation [19]. Several studies on profiling localized mRNAs in cells suggest their function in a variety of cellular physiology [20,21,22,23]. However, the role of localized ncRNAs is not studied actively despite available high throughput sequencing data sets.

Here, we report role of nuclear genome-encoded mitochondrial ncRNA, *RPPH1* RNA (Ribonuclease P RNA component H1) [24], in cellular senescence. The molecular function of *RPPH1* RNA has been less clear beyond its canonical role in the 5′ maturation of precursor transfer RNAs (pre-tRNAs) [25]. We observed *RPPH1* RNA is overexpressed in senescent fibroblasts and accumulates in its mitochondria. We identified *MLC1* and *CCR7* mRNA as target mRNAs of *RPPH1* RNA based on in silico analysis and confirm the biochemical interaction between those mRNAs and *RPPH1* RNA using MS2-TRAP (MS2-tagged RNA affinity purification) [26]. Luciferase reporter assay suggested that *RPPH1* RNA increases stability of its target mRNAs. One of the RBPs, AUF1, maintains *RPPH1* RNA level in cytoplasm to keep *MLC1* and *CCR7* mRNAs stable. Upon *RPPH1* RNA accumulation in mitochondria of senescent fibroblasts, these mRNAs are labile to degradation. All in all, our study revealed that *RPPH1* RNA functions to increase the stability of target mRNAs with partial complementarity, which might work to prevent proliferating fibroblasts from cellular senescence.

## 2. Results

### 2.1. Expression Changes of Mitochondrial RNAs in Cellular Senescence

**Figure 1:**
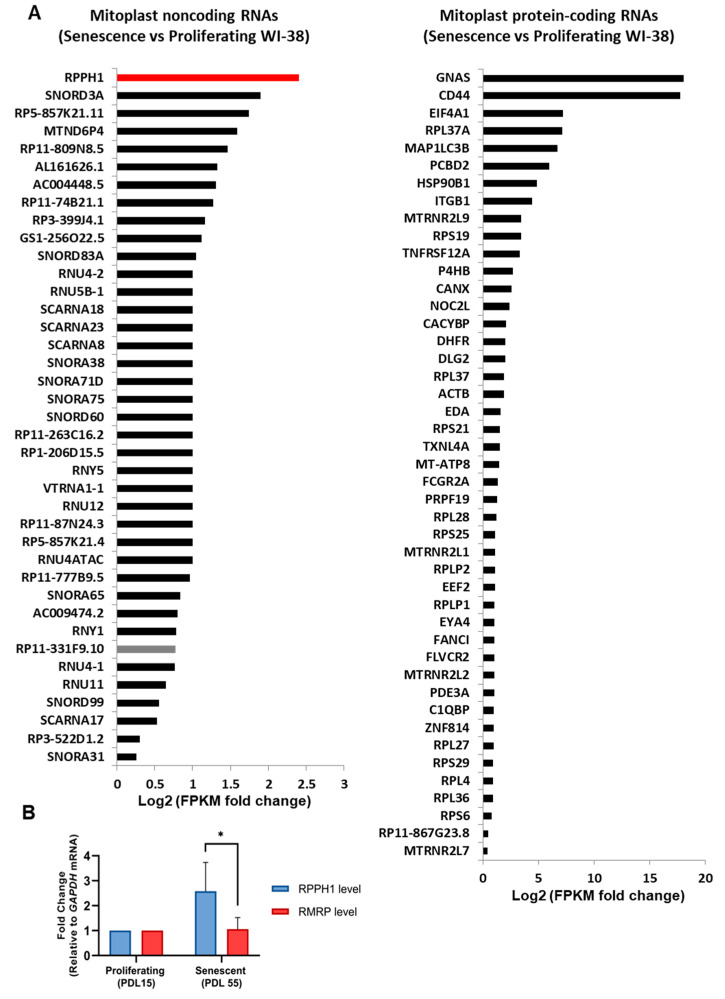
Mitochondrial noncoding and protein-coding RNAs differentially expressed in proliferating and senescent fibroblasts. (**A**) Mitochondrial noncoding RNAs and mRNAs with FPKM log2 fold change in PDL 15 (Proliferating) and PDL 55 (Senescent) WI-38 cells. (**B**) RT-qPCR analysis of *RPPH1* and *RMRP* RNAs normalized with *GAPDH* mRNA. RNA was quantified after reverse transcription and qPCR from whole cell. Data in (**B**) is average +/− S.D of independent three experiments. Asterisks denote statistical significance (Student’s *t* test): * *p* ≤ 0.05.

Recent advances in RNA sequencing technology have enabled annotation of human mitochondrial RNAs [21]. In human 143B cells, mitochondrial transcripts originating from nuclear or mitochondrial genomes are characterized. Unexpectedly, a variety of RNAs are aligned to nuclear genomes expressing protein-coding RNAs and ncRNAs. In order to determine the change of mitochondrial transcripts during cellular senescence, we first surveyed of previously published datasets of RNA-seq to extract information regarding differentially expressed mitochondrial transcripts during replicative cellular senescence. To this end, we incorporated RNA-seq data from mitoplasts [21] with RNA-seq data from proliferating (PDL15) and senescent (PDL55) WI-38 cells [27,28]. Since the mitoplasts were prepared by stripping of mitochondrial outer membrane copurifying RNA contaminants can be eliminated in the mitoplasts RNA-seq [21]. Our analysis has shown that 39 ncRNAs are differentially expressed in mitoplasts of senescent fibroblasts compared to that of proliferating fibroblasts (Figure 1A, left). Similarly, 45 protein-coding RNAs were fluctuated in their expression in mitoplasts during cellular senescence (Figure 1A, right). Our results from the publicly available data sets suggest that the expression levels of mitochondrial transcripts fluctuate during senescence.

### 2.2. A Senescence-Associated Mitochondrial Localization of RPPH1 RNA Is Modulated by AUF1

**Figure 2:**
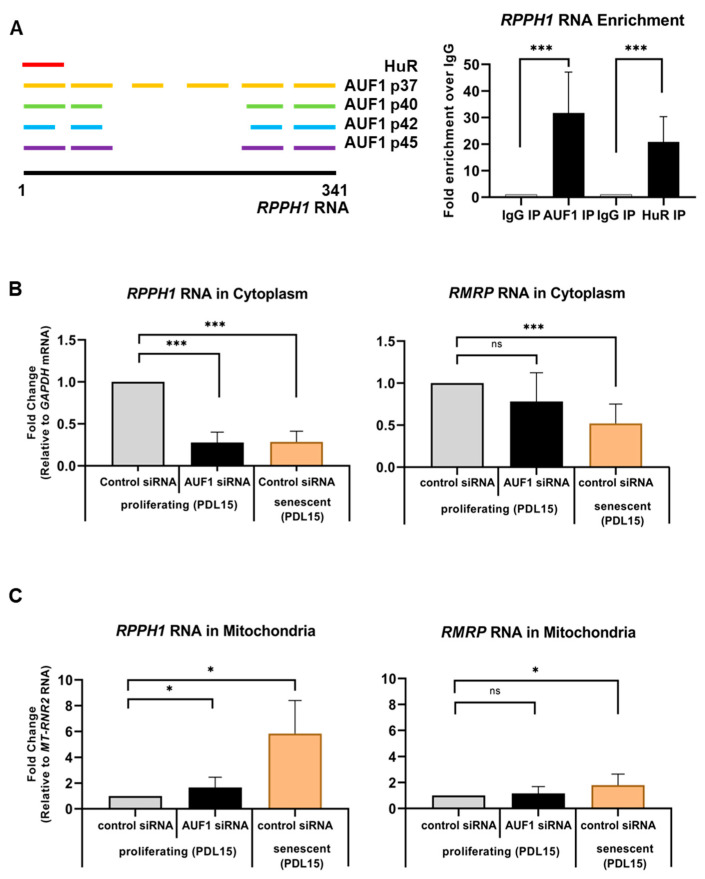
Mitochondrial accumulation of RPPH1 RNA is regulated by AUF1. (**A**) AUF1 and HuR binding sites on *RPPH1* RNA identified from PAR-CLIP analysis. RIP analysis showing relative enrichment of *RPPH1* RNA in AUF1 or HuR immunoprecipitation compared to IgG control in WI-38 cells as measured by whole cell RT-qPCR and normalized to *GAPDH* mRNA levels. (**B**) Relative expression of *RPPH1* (left) and *RMRP* (right) RNAs in cytoplasm of proliferating (control or AUF1-silenced) or senescent WI-38 cells measured by RT-qPCR normalized with *GAPDH* mRNA. (**C**) Relative expression of *RPPH1* (left) and *RMRP* (right) RNAs in mitochondria of proliferating (control or AUF1-silenced) or senescent WI-38 cells measured by RT-qPCR normalized with 16S rRNA, *MT-RNR2*. Data in (**A**–**C**) are average +/− S.D of independent three experiments. Asterisks denote statistical significance (Student’s *t* test): ns *p* > 0.05, * *p* ≤ 0.05, *** *p* ≤ 0.001.

Among many transcripts in our analysis, we focused on the most upregulated mitochondrial RNA, *RPPH1* RNA, in senescent fibroblasts (Figure 1A). *RPPH1* RNA is one of the RNA components in ribonuclease P which cleaves tRNA precursors during 5′ maturation [25]. While we observed that total *RPPH1* RNA is overexpressed in senescent fibroblasts, another RNA component of RNase P, *RMRP* RNA, did not fluctuate significantly in cellular senescence (Figure 1B). In order to identify protein factors contributing to *RPPH1* RNA overexpression in senescent fibroblasts, we examined publicly available CLIP data base and found 21 RBPs including AUF1 and HuR (starbase v2.0) [28]. Within 341 nt of full length *RPPH1* RNA, there are 1 HuR site, 6 AUF1 p37 sites, 4 p40 sites, 4 p42 sites, and 4 p45 sites (Figure 2A, left). We confirmed the interaction between *RPPH1* RNA and AUF1 by Ribonucleoprotein Immunoprecipitation (RIP) followed with qPCR analysis (Figure 2A, right). Although AUF1 is generally known to function as a RNA decay factor, depletion or overexpression of AUF1 did not affect steady state level of *RPPH1* and *RMRP* RNAs (Figure S1). Similarly, modulation of HuR expression did not affect steady state level of *RPPH1* and *RMRP* RNAs either (Figure S2A,B). Our results revealed that *RPPH1* RNA is a target RNA of AUF1 and HuR, however, these RBP do not affect steady state levels of *RPPH1* RNA. When we measure the stability of *RPPH1* RNA upon AUF1 depletion, its stability did not change significantly (Figure S2C). Since *RPPH1* RNA is localized in nucleus, cytoplasm, and mitochondria [24,29], we asked if AUF1 affects *RPPH1* RNA subcellular localization. Interestingly, AUF1 silencing reduced steady state levels of *RPPH1* RNA in cytoplasm whereas the mitochondrial *RPPH1* RNA levels were increased in proliferating HDFs (PDL15). Interestingly, reduced cytosolic *RPPH1* levels in conjunction with increased mitochondrial *RPPH1* levels were observed in senescent HDFs (PDF55) when compared to proliferating HDFs (PDL15) (Figure 2B,C, left). The possible involvement of AUF1 in shifting *RPPH1* RNA from the cytoplasm to the mitochondria is supported by the observation that AUF1 levels are reduced during cellular senescence (Figure S2D). In contrast, the mitochondrial and the cytoplasmic distribution of *RMRP* RNA did not change dramatically after AUF1 depletion in proliferating HDFs. Moderate changes of *RMRP* RNA distribution between proliferating—and senescent HDFs were observed (Figure 2B,C, right). Validation of subcellular fractionation was confirmed by western blot analysis in that ATP5A used as a mitochondria marker and GAPDH used as a cytoplasmic marker (Figure S2E). These results demonstrate AUF1 is required for optimal localization of *RPPH1* RNA in cytoplasm and mitochondria of proliferating fibroblasts.

### 2.3. Replicative Cellular Senescence Is in Part Attributed to Mitochondrial RPPH1 Overexpression

**Figure 3:**
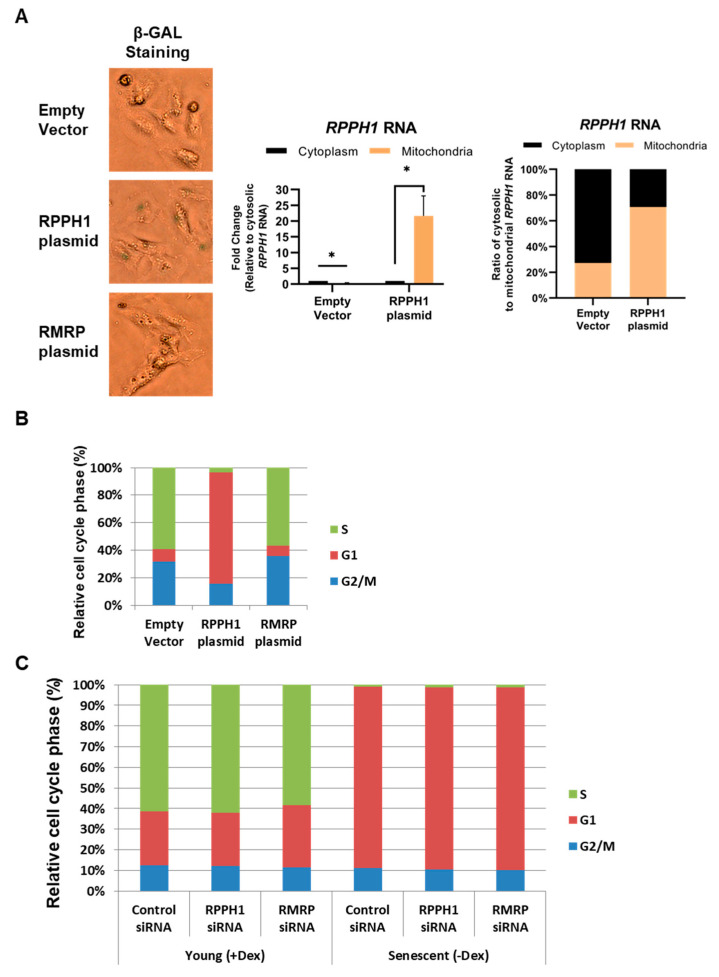
*RPPH1* RNA overexpression promotes cellular senescence. (**A**) Acidic β-galactosidase staining (left) after 7 days of Empty Vector, RPPH1, or RMRP plasmid transfection in WI-38 cells. Mitochondrial localization of ectopically expressed *RPPH1* RNA was confirmed by RT-qPCR after mitochondrial isolation (middle). Proportion of cytosolic *RPPH1* RNA over mitochondrial *RPPH1* after transfection of either Empty vector or RPPH1 plasmid (right). (**B**) Proportion of cell division cycle stages after transfection of Empty Vector, RPPH1, or RMRP plasmid. (**C**) Cell cycle analysis of proliferating (+Dex) and senescent (−Dex) IDH4 cells after transfection of Control, RPPH1, or RMRP siRNA. Data are representative of independent three experiments. Data in (**A**) is average +/− S.D of independent three experiments. Asterisks denote statistical significance (Student’s *t* test): * *p* ≤ 0.05.

Overexpression and increased mitochondrial localization of *RPPH1* RNA in senescent fibroblasts suggest its possible function in the promotion of cellular senescence. We tested this possibility after overexpression of Empty vector, RPPH1, or RMRP plasmid in proliferating fibroblasts (WI-38, PDL 15). Seven days after transfection, senescence associated β-galactosidase staining revealed that *RPPH1* RNA overexpression promotes activation of acidic β-galactosidase as a marker of cellular senescence (Figure 3A, left). We observed that ectopically expressed *RPPH1* RNA are significantly expressed in the mitochondria of proliferating HDFs (Figure 3A, middle), and proportion of cytosolic *RPPH1* RNA over mitochondrial *RPPH1* was decreased in *RPPH1* overexpression compared to control vector (Figure 3A, right). Measurement of cell division cycle by Propidium Iodide (PI) and Flow Cytometry uncover that *RPPH1* RNA overexpression arrested proliferating fibroblasts (WI-38, PDL 15) in G1 stage whereas *RMRP* RNA overexpression did not affect it (Figure 3B). In contrast to *RPPH1* RNA overexpression in senescent fibroblasts and its sufficiency to promote cellular senescence in WI-38 cells, *RPPH1* or *RMRP* RNA was not required for cellular senescence induced by dexamethasone removal in IDH4 fibroblasts (Figure 3C), implying that *RPPH1* RNA does not come into play in hormone-associated senescence. These results demonstrate that mitochondrial *RPPH1* RNA promotes replicative cellular senescence in WI-38 fibroblasts.

Next, we asked the consequences of *RPPH1* RNA distribution change in proliferating—and senescent fibroblasts. LncRNAs have been widely implicated in post-transcriptional gene regulation and one of proposed model is to interact with target mRNAs based on partial complementarity [9]. 

**Figure 4:**
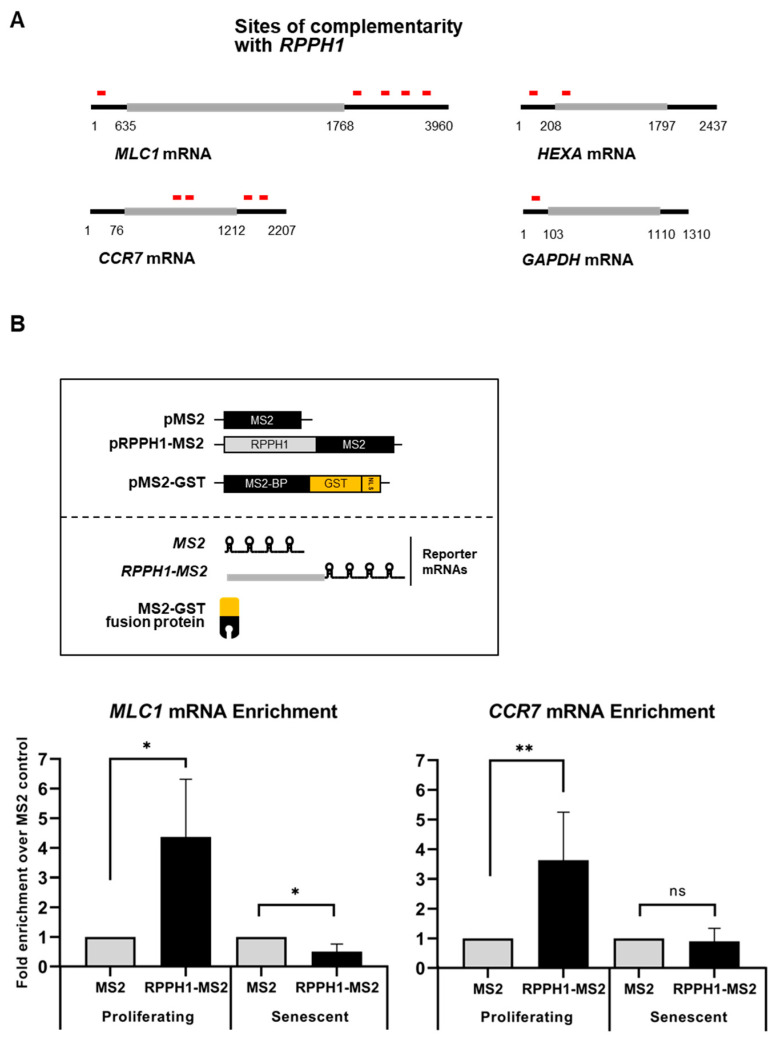
*RPPH1* RNA interacts with its target mRNAs using complementary sequences. (**A**) Sites of complementarity with *RPPH1* RNA on *MLC1*, *HEXA*, *CCR7*, and *GAPDH* mRNAs. (**B**) MS2-GST pull down analysis to detect *MLC1* and *CCR7* mRNAs in proliferating and senescent fibroblasts. *MLC1* (left) and *CCR7* (right) mRNA pull-down enrichment comparing proliferating and senescent cells as measured by RT-qPCR normalized with *GAPDH* mRNA. Data in (**B**) are average +/− S.D of independent three experiments. Asterisks denote statistical significance (Student’s *t* test): ns *p* > 0.05, * *p* ≤ 0.05, ** *p* ≤ 0.01.

**Figure 5:**
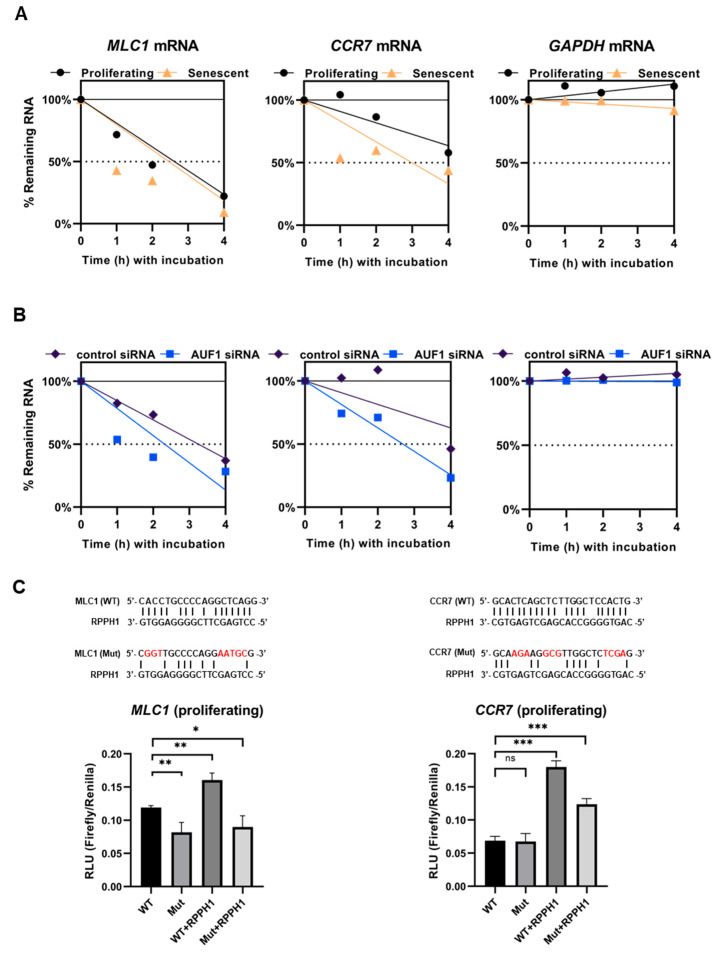
Target mRNAs of *RPPH1* RNA are destabilized in cellular senescence. (**A**) Stability of *MLC1*, *CCR7*, and *GAPDH* mRNAs in proliferating or senescent WI-38 cells. (**B**) Stability of *MLC1*, *CCR7*, and *GAPDH* mRNAs in proliferating WI-38 cells after transfection of Control or AUF1 siRNA (~48 h) as measured by RT-qPCR. (**C**) Luciferase reporter assay with constructs bearing putative *RPPH1* binding sites. Reporters carrying an indicate site (sequence above bar-graphs) were co-transfected with RPPH1 expression vector, and luciferase activity was measured. Data in (**A**–**C**) are representative of three independent experiments. Asterisks denote statistical significance (Student’s *t* test): ns *p* > 0.05, * *p* ≤ 0.05, ** *p* ≤ 0.01, *** *p* ≤ 0.001.

**Figure 6:**
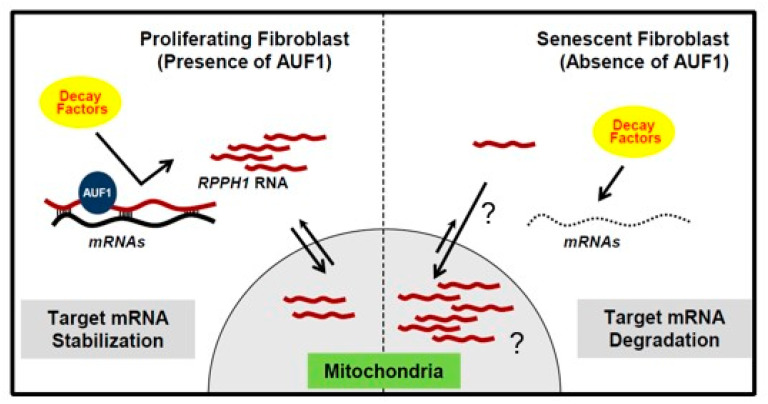
A proposed model of *RPPH1* RNA function on target mRNAs’ destabilization during cellular senescence. With *RPPH1* localized in the mitochondria during senescence, target mRNAs’ association with *RPPH1* is lowered, and thus they are more subject to cytoplasmic decay. AUF1 facilitates the cytoplasmic retention of *RPPH1* RNA, however it is unknown how AUF1-free *RPPH1* RNA move to the mitochondria. The mitochondrial accumulated *RPPH1* might possess other unknown function beyond target mRNA protection which also contribute to cellular senescence.

First, we investigated mRNAs having complementarity with *RPPH1* RNA as performed previously [30]. Our BLAST-based search revealed that three mRNAs (*MLC1*, *HEXA*, and *CCR7* mRNAs) have partial complementarity with *RPPH1* RNA above threshold (E-Value < 2, Identity > 75%, Nucleotide Match > 13) (Figure 4A) (Table S1). Prediction of partial complementarity was tested in MS2-tagged RNA affinity purification assay [26], confirming that *MLC1* and *CCR7* mRNAs are biochemically associated with *RPPH1* RNA in proliferating fibroblasts but not senescent ones (Figure 4B). Emerging roles for intermolecular lncRNA-mRNA interactions in mRNA metabolism have been proposed [31]. In addition, we observed that *MLC1* and *CCR7* mRNAs are destabilized in senescent fibroblasts where *RPPH1* RNA is less abundant in cytoplasm (Figure 5A). We also analyzed the published RNA-seq datasets from the diverse models of senescence [32], and found that the levels of *MLC1* and *CCR7* mRNA are declined during replicative and irradiation-mediated senescence in IMR90 cells in conjunction with modestly decreased *AUF1* mRNA level and increased *p16* and *p21* mRNA (senescent markers) (Figure S3A–I). However, this trend is not observed before and after senescence by diverse triggers such as replicative exhaustion, exposure to irradiation or doxorubicin or an oncogene expression in human endothelial cells (HUVEC, HAEC) and human diploid fibroblasts (WI-38) (Figure S3A–I). Similarly, mRNAs targeted by *RPPH1* RNA are unstable after AUF1 depletion in the condition when *RPPH1* RNA accumulates in mitochondria (Figure 5B). In order to ensure that regulation of target mRNAs stability indeed derived from the interaction via the putative binding site (Table S1), a luciferase reporter plasmid was generated, containing the *RPPH1* binding site. Next, the reporter plasmid was co-transfected with the RPPH1 overexpression vector, into proliferating HDFs resulting in the increased luciferase activity. However, a mutant reporter plasmid where the binding site were disrupted suppressed luciferase activity (Figure 5C). These results demonstrate that *RPPH1* RNA interacts with its target mRNAs to inhibit their decay. Taken together, our results suggest that the role of *RPPH1* RNA in the cytoplasm promoting their target mRNAs stability is dampened in senescent fibroblasts possibly due to downregulation of AUF1 during cellular senescence (Figure 6).

## 3. Discussion

Our findings unveil the role of senescence-associated mitochondrial *RPPH1* RNA in stabilization of target mRNAs. We identified protein-coding RNAs and ncRNAs whose expression changes in replicative senescence (Figure 1). Among many mitochondrial RNAs, *RPPH1* RNA interacts with RBP AUF1 resulting in its optimal cytoplasmic and mitochondrial distribution (Figure 2). Ectopic expression of *RPPH1* RNA promotes replicative cellular senescence while it is dispensable for hormone-associated senescence (Figure 3). However, it remains elusive how ectopic *RPPH1* RNA moves to the mitochondria and this leads to override the function of endogenous *RPPH1* RNA in the cytoplasm in proliferating fibroblasts. Partial complementarity of *RPPH1* RNA with its target mRNAs prevents their decay in proliferating fibroblasts (Figure 4 and Figure 5). Taken together, our data revealed that *RPPH1* RNA promotes cellular senescence by destabilization its target mRNAs in the cytoplasm (Figure 6).

### 3.1. Senescence-Associated Mitochondrial Noncoding RNAs

Recently a variety of noncoding RNAs have been sequenced, aligned to genomes, annotated, and characterized in their function. Besides ncRNA from total RNAs, ncRNAs in organelles have also been sequenced and studied [21,22,23]. While previous studies of mitochondrial transcriptome revealed that mitochondrial transcripts are originated from both mitochondrial and nuclear genome, our study characterized mitochondrial *RPPH1* RNA originating from nuclear genome. In addition, we coupled mitochondrial RNA sequencing data with senescence RNA sequencing data [27,28] in order to characterize senescence-associated mitochondrial RNAs. Despite our data on mitochondrial ncRNAs differentially expressed in proliferating and senescent fibroblasts, direct analysis of RNA sequencing with mitochondrial fractions is desperately required. Since mitochondria have very similar characteristics to the nucleus in terms of double-membrane structure, more careful approaches on pure mitochondrial purification is expected, as performed previously [21]. Complexity of mitochondrial transcriptome and removal of mitochondrial ribosomal RNA also contribute to the quality of RNA sequencing data significantly. Altogether, our study provided the first trial on characterization of nuclear genome-encoded mitochondrial ncRNAs in cellular senescence.

### 3.2. Senescence-Induced RPPH1 RNA Accumulation in Mitochondria

Senescence triggers dramatic changes in cellular physiology [33] including dramatic inhibition of transcription, RNA decay, and mRNA translation [34,35]. While the transcription rate is decreased dramatically, overall steady state levels of mRNAs do not change significantly during cellular senescence [36]. Recent studies revealed that senescence-associated secretory phenotype (SASP) is regulated by mTOR-mediated translation [37,38]. These results suggest the importance of post-transcriptional gene regulation including mRNA decay and translation in cellular senescence. Our study on senescence-associated mitochondrial ncRNAs revealed that *RPPH1* RNA is differentially distributed in the cytoplasm and the mitochondria during cellular senescence. We have shown that *RPPH1* RNA accumulates in mitochondria of senescent fibroblasts compared to proliferating ones (Figure 2). In addition, we also demonstrated that AUF1 depletion, a condition when cellular senescence is promoted, also triggered *RPPH1* RNA accumulation in mitochondria. Although the exact mechanism of how *RPPH1* RNA is transported to or out of mitochondria should be studied further, this result implicates the significant shifting of *RPPH1* RNA from the cytoplasm to the mitochondria is associated with cellular senescence. A recent report demonstrates that HuR may regulate the cytoplasmic and mitochondrial localization of *RMRP* RNA [39]; however, its subcellular localization is not influenced significantly by AUF1 (Figure 2B,C) implying that there is a distinct mechanism for *RPPH1* RNA distribution which might be regulated by AUF1, but not HuR despite our result showing the interaction between *RPPH1* RNA and HuR (Figure 2A). In sum, our data demonstrate a change in *RPPH1* RNA distribution in cytoplasm and mitochondria during cellular senescence.

### 3.3. Target mRNA Stabilization by Partial RNA Complementarity

Long noncoding RNAs (lncRNAs) modulate post-transcriptional gene regulation [31,40]. Association of lncRNAs with target RNAs including mRNAs and microRNAs occurs through its complementary sequences. Interaction of lncRNA with target mRNAs promotes their decay [40] or suppresses their degradation [41]. LncRNA binding to miRNA triggers its decay [30,31] while lncRNA also titrates miRNA concentration [40]. In our study, we revealed that *RPPH1* RNA could associate with its target mRNAs using partial complementarity. Since most of the complementarity regions are involved in intramolecular base pairing in *RPPH1* RNA, the interaction between *RPPH1* RNA and its target mRNAs could occur indirectly [42]. Even though it is not clear how target mRNAs are degraded after dissociation from *RPPH1* RNA, possible mechanisms can be to increase a chance of target mRNAs exposing to mRNA decay machineries (Figure 5). These include 5′ decapping/5′-to-3′ decay, deadenylation/3′-to-5′ decay, endonucleolytic cleavage, and miRNA-mediated degradation. Decreased interaction of *RPPH1* RNA with target mRNAs in senescent fibroblasts demonstrates that *RPPH1* RNA prevents undesirable decay of target mRNAs in proliferating fibroblasts. It is also plausible that *RPPH1* RNA is associated with other mitochondrial mRNAs which were screened out by the threshold setting we used. In this circumstance, mitochondrial localization of *RPPH1* RNA might impede the fine-tuning of expression of mitochondrial proteins resulting in dysregulated mitochondrial integrity. It has not been characterized the functions of *MLC1* (Modulator of VRAC current 1) and *CCR7* (C-C motif chemokine receptor 7) in aging and cellular senescence. Further studies should follow to investigate whether target mRNAs of *RPPH1* RNA (*MLC1*, *CCR7*, and *HEXA* mRNAs) are required for promotion of cellular senescence. Since our data showed that the AUF1-*RPPH1*-*MLC1*/or *CCR7* mRNA axis is not implicated in all of the diverse models of senescence (Figure S3A–I), we cannot rule out the possibility that cellular senescence induced by mitochondrial accumulation of *RPPH1* RNA may be influenced by impaired tRNA 5′ processing, not necessarily due to direct *RPPH1* RNA-mRNA interactions. Another caveat is that accumulated *RPPH1* RNA in the mitochondria might lead to dysregulated function of the mitochondria via interaction with mitochondrial RNAs or proteins which need to be addressed in future study. In sum, this study unveiled the function of *RPPH1* RNA in cellular senescence with its function in target mRNA stabilization.

## 4. Material and Methods

### 4.1. Cell Culture, Transfection, Small Interfering RNAs, and Plasmids

Human fibroblasts WI-38 (Coriell Cell Repositories, Camden, NJ, USA) and IDH4 (a gift from Dr. J.W. Shay, UT Southwestern) were cultured in DMEM (Invitrogen, Carlsbad, CA, USA) supplemented with 10% (*v*/*v*) fetal bovine serum (Hyclone, Logan, Utah, USA) and 1% penicillin/streptomycin (Lonza, Basel, Switzerland). All cultured cells were maintained at 37 °C in humid condition with 5% CO_2_. Cells were transfected (Lipofectamine 2000, Invitrogen, Carlsbad, CA, USA) with siRNAs (20 nM) [Control (UUCUCCGAACGUGUCACGU), AUF1 (AAGAUCCUAUCACAGGGCGAU), RPPH1 (CUCCCAUGUCCCUUGGGAAGGUC), HuR (CGUAAGUUAUUUCCUUUAA), or RMRP (GGCUACACACUGAGGACUC) siRNA]. 1–2 μg of plasmids that expressed AUF1 isoforms, RPPH1, or RMRP were transfected using Lipofectamine 2000 according to the manufacturer’s protocol. Transfected cells were generally analyzed 48 h later.

### 4.2. Western Blot Analysis

Western blot analysis was performed as reported previously [30]. Briefly, total cell lysates were prepared in RIPA buffer (10 mM Tris–HCl, pH 7.4, 150 mM NaCl, 1% (*v*/*v*) Nonidet P-40, 1 mM EDTA, and 0.1% (*w*/*v*) SDS). Protein samples were separated on 10% SDS-polyacrylamide (SDS-PAGE) gels, and transferred onto Nitrocellulose membranes (Invitrogen iBlot Stack, Carlsbad, CA, USA). The membranes were incubated with a specific primary antibody in Tris-buffered saline (TBS) containing 0.05% Tween 20 (TBS-T) and 5% nonfat dry milk at 4 °C for overnight. After three washes with TBS-T, the blots were incubated with horseradish peroxidase-conjugated secondary antibodies (GE Healthcare, Chicago, IL, USA) for 1 h at room temperature, immunoblots were visualized using Amersham ECL Prime Western Blotting Detection Reagent (GE Healthcare, Chicago, IL, USA). The following antibodies were purchased from Millipore (Burlington, MA, USA): anti-AUF1, Anti-HuR, anti-beta-Actin, anti-GAPDH, and anti-ATP5A were purchased from Santa Cruz Biotechnology (Dallas, TA, USA).

### 4.3. RNP Analysis

Immunoprecipitation (IP) of endogenous RNP complexes (RIP analysis) from whole-cell extracts was performed as described previously [30]. Briefly, the total cells were lysed in the cell lysis buffer composed of 20 mM Tris-HCl at pH 7.5, 100 mM KCl, 5 mM MgCl_2_ and 0.5% NP-40 for 10 min on ice and centrifuged at 10,000× *g* for 15 min at 4 °C. The lysates were incubated with 1 µg of antibodies against AUF1, HuR (Santa Cruz Biotechnology, Dallas, TA, USA), or with control IgG (Santa Cruz Biotechnology, Dallas, TA, USA) conjugated with protein A-Sepharose beads for 1 h at 4 °C. After the beads were washed five times with NT2 buffer composed of 50 mM Tris-HCl at pH 7.5, 150 mM NaCl, 1 mM MgCl_2_ and 0.05% NP-40, the beads were incubated with 20 units of RNase-free DNase I for 15 min at 37 °C. Then the beads were further incubated with 0.1% SDS/0.5 mg/mL Proteinase K for 15 min at 55 °C. The beads-associated RNA was extracted with acidic phenol and further examined by reverse transcription (RT)-quantitative polymerase chain reaction (qPCR) analysis using the primers listed (Table S1). MS2 pull-down was performed as described previously [26]. Briefly, a plasmid expression *RPPH1* RNA was used to construct plasmid pRPPH1-MS2 which expresses chimeric RNA (*RPPH1*-*MS2* hairpins). WI-38 cells were co-transfected with pRPPH1-MS2 and pMS2-GST which expresses a fusion protein that contained a glutathione-S-transferase domain fused to a domain that recognizes MS2 hairpins. Forty-eight hours later, cells were lysed with the NT2 buffer aforementioned, then mRNAs associated with the chimeric RNA were pulled down by using glutathione (GSH)-coated beads. Following extraction of RNA from the beads, we employed RT-qPCR analysis to confirm the biochemical interaction between *RPPH1* RNA and target candidate mRNAs observed from the BLAST-based search. Normalization of RIP results was carried out by quantifying in parallel the relative levels of *GAPDH* mRNA. These abundant RNAs are nonspecific contaminants present in the IP components (microfuge tube, beads, etc.).

### 4.4. RNA Quantification by qPCR

Total RNA of cells was isolated by Trizol (Invitrogen, Carlsbad, CA, USA) according to the manufacturer’s protocol. Acidic phenol (Ambion, Carlsbad, CA, USA) was used to extract RNA for RIP analysis. Isolated RNA was reverse transcribed using random hexamers and reverse transcriptase (Maxima Reverse Transcriptase, Fermentas, Waltham, MA, USA). After RT, cDNAs were assessed via qPCR analysis with SYBR green master mix (Kapa Biosystems, Salt River Cape Town, South Africa), gene-specific primers listed (Table S2), and an Applied Biosystems 7300 instrument. Thermal cycling conditions were as follows: 95 °C for 10 min, followed by 50 cycles of 95 °C for 15 s and 60 °C for 1 min. Relative quantities of RNAs were calculated using ΔΔCt method and normalized Ct values to those of *GAPDH* mRNA, *MT-RNR2* RNA, and *18S* rRNA. All oligonucleotides are displayed in Table S2.

### 4.5. Subcellular Fractionation

Cytosolic and mitochondrial fractions were collected as described in Manufacturer’s Protocol (Mitochondria Isolation Kit, PIERCE, Rockford, IL, USA). Briefly, cells were lysed with Reagent A for 2 min on ice, Reagent B for 5 min on ice with vortexing every minute, and Reagent C, then the resulting lysates were centrifuged at 700× *g* for 10 min at 4 °C. The supernatant was used for the cytosolic fraction. The pellets were centrifuged at 12,000× *g* for 15 min at 4 °C, supernatant was removed, pellets were washed with Reagent C, centrifuged at 12,000× *g* for 5 min at 4 °C to get mitochondrial fraction. Subcellular fractionation was verified by western blot analysis.

### 4.6. Bioinformatic Analysis of RPPH1 RNA Interaction Sites with mRNAs

We used BLAST (http://blast.ncbi.nlm.nih.gov/) to identify local regions of sequence similarity between *RPPH1* RNA and *MLC1*, *CCR7*, *HEXA*, and *GAPDH* mRNA (NM_002046.3). The similarity regions with E-Value < 2, Identity > 75%, Nucleotide Match > 13 matching to the reverse complementary sequence of *RPPH1* RNA were selected as and considered as possible interaction regions through base-paring between *RPPH1* RNA and each mRNA. Table S1 lists the putative interaction regions identified.

### 4.7. Cellular Senescence Assay

WI-38 cells were cultured from population doubling level (PDL) 15 to reach PDL 55 after splitting the cells into half upon confluency. IDH4 cells, in which the expression of SV40 large-T antigen is regulated by a steroid-inducible promoter, were cultured in the presence of 1 mM Dexamethasone (Dex) to suppress cellular senescence and promoted proliferation. Suppression of SV40 large-T antigen in IDH4 to induce senescence was mediated by Dex removal from the IDH4 culture media (charcoal-stripped serum). Cellular senescence was examined 7 days later after transfection. Senescence-associated β-galactosidase activity was assessed by senescence β-galactosidase staining kit (Cell Signaling Technology, Danvers, MA, USA) according to the manufacturer’s instructions. The percentages of G1, S and G2/M cells was determined by standard fluorescence-activated cell sorting analysis.

### 4.8. Luciferase Reporter Assay

Luciferase reporter constructs bearing the putative binding sites in *MLC1* and *CCR7* mRNAs interacting with *RPPH1* (Table S1) were cloned into pmiR-GLO plasmid (Promega, Madison, WI, USA) right after the firefly luciferase gene. The pmiR-GLO plasmid was digested with XbaI (New England Biolabs, Ipswich, MA, USA) and gel purified. To insert DNA, annealed oligos containing the *MLC1*, *CCR7* mRNA site interacting with *RPPH1* (37 °C for 30 min, 95 °C ramped down to 25 °C at 5 °C/min), and ligated into the digested pmiR-GLO vector. Positive colonies were confirmed by PCR, and the construction of pmiR-GLO-MLC1 and pmiR-GLO-CCR7 vectors were verified by sequencing. Generated luciferase reporter plasmids were co-transfected with pRL-null vector using Fugene HD transfection reagent (Promega, Madison, WI, USA) according to the manufacturer’s protocol. Transfected cells were generally analyzed 48 h later. Luciferase activities were measured using a DualGlo Luciferase Assay Kit (Promega, Madison, WI, USA) according to the manufacturer’s protocol.

### 4.9. Statistical Analysis

Quantitative data are presented as the mean ± SD, unless otherwise indicated. Differences between means were evaluated using Student’s unpaired *t* test. Results were considered statistically significant at ns *p* > 0.05, * *p* ≤ 0.05, ** *p* ≤ 0.01, *** *p* ≤ 0.001.

## Data Availability

Not applicable.

## Supplementary Materials

The following are available online at https://www.mdpi.com/1422-0067/22/2/782/s1, Figure S1: AUF1 does not influence steady state levels of *RPPH1* and *RMRP* RNA, Figure S2: Additional analysis of *RPPH1* RNA, *HuR* mRNA and *RMRP* RNA, Figure S3: Relative expression of senescence-related RNAs in various cell types and senescence inducers. Table S1: Putative regions of interaction of human *RPPH1* RNA (subject) with human *HEXA* mRNA, *CCR7* mRNA, *MLC1* mRNA, and *GAPDH* mRNA (query), Table S2: List of primers used in this study.
