# Supplementary material for: Accumulation of Mitochondrial RPPH1 RNA Is Associated with Cellular Senescence
Source: Int J Mol Sci. 2021 Jan 14;22(2):782. doi: 10.3390/ijms22020782 (PMC7828772; doi:10.3390/ijms22020782)
Supplement: Supplementary file 1 [file ijms-22-00782-s001.pdf]

Supplemental Table 1. Putative Regions of interaction of human *RPPH1* RNA (subject) with human *HEXA* mRNA, *CCR7* mRNA, *MLC1* mRNA, and *GAPDH* mRNA (query).

<table>
<tr><th>Target mRNA</th><th>Matched Sequence</th><th>Identity (%)</th><th>E-Value</th></tr>
<tr><td></td><td></td><td></td><td></td></tr>
<tr><td>HEXA mRNA</td><td>Query 273 CCTCTGGCCCTGGCCTCAGAACTTCCAAA 301
||||||||||| | |||||| ||||| ||
Sbjct 99 CCTCTGGCCCTAGTCTCAGACCTTCCCAA 71</td><td>25/29(86%)</td><td>2e-05</td></tr>
<tr><td>HEXA mRNA</td><td>Query 102 AGAGCCGCCTCTGG 115
|| |||||||||||
Sbjct 106 AGGGCCGCCTCTGG 93</td><td>13/14(93%)</td><td>0.35</td></tr>
<tr><td></td><td></td><td></td><td></td></tr>
<tr><td>CCR7 mRNA</td><td>Query 1930 GCACTCAGCTCTTGGCTCCACTG 1952
||||||||||| |||| ||||||
Sbjct 44 GCACTCAGCTCGTGGCCCCACTG 22</td><td>21/23(91%)</td><td>5e-05</td></tr>
<tr><td>CCR7 mRNA</td><td>Query 1543 CAAGGGGCGTGGGAGTGG 1560
|||||| | |||||||||
Sbjct 73 CAAGGGACATGGGAGTGG 56</td><td>18/18(89%)</td><td>0.026</td></tr>
<tr><td>CCR7 mRNA</td><td>Query 783 GCTGGCCATGAGCTTCTGTT 802
||||||| |||| ||||||
Sbjct 262 GCTGGCCGTGAG--TCTGTT 245</td><td>17/20(85%)</td><td>0.31</td></tr>
<tr><td>CCR7 mRNA</td><td>Query 655 CTCCTGTACAGT--GACCTCCAGAGGAGC 681
|||||| |||| |||||| | |||||
Sbjct 183 CTCCTGCCCAGTCTGACCTCGCGCGGAGC 155</td><td>22/29(76%)</td><td>1.1</td></tr>
<tr><td></td><td></td><td></td><td></td></tr>
<tr><td>MLC1 mRNA</td><td>Query 2153 GGGTCC-CTGCGTCTCCTGCCCACTCTGACC 2182
|||||| | || ||||||||||| |||||||
Sbjct 196 GGGTCCACGGCATCTCCTGCCCAGTCTGACC 166</td><td>27/31(87%)</td><td>3e-05</td></tr>
<tr><td>MLC1 mRNA</td><td>Query 2264 CACCTGCCCCAGGCTCAGG 2282
||||| ||| | |||||||
Sbjct 137 CACCTCCCCGAAGCTCAGG 119</td><td>16/19(84%)</td><td>0.56</td></tr>
<tr><td>MLC1 mRNA</td><td>Query 3676 CACCTCCCTGCAGCT 3690
|||||||| | ||||
Sbjct 137 CACCTCCCCGAAGCT 123</td><td>13/15(87%)</td><td>2.0</td></tr>
<tr><td>MLC1 mRNA</td><td>Query 2423 GTCCACGCCGGGTCGCCTGCCCTGTCT 2449
||||||| | || ||||||| ||||
Sbjct 194 GTCCACGGCA--TCTCCTGCCCAGTCT 170</td><td>21/27(78%)</td><td>2.0</td></tr>
<tr><td>MLC1 mRNA</td><td>Query 28 AAAGGGGGCAGCAGGACCGGGCCC 51
||||| |||| | | ||||||||
Sbjct 232 AAAGGAGGCATCCG--CCGGGCCC 211</td><td>19/24(79%)</td><td>2.0</td></tr>
<tr><td></td><td></td><td></td><td></td></tr>
<tr><td>GAPDH mRNA</td><td>Query 42 GGCGGGGTCCGAGTCACCGCCTGCC 66
|||||||||| | || | ||||||
Sbjct 200 GGCGGGGTCCACGGCATCTCCTGCC 176</td><td>20/25(80%)</td><td>0.058</td></tr>
<tr><td></td><td></td><td></td><td></td></tr>
</table>

Supplemental Table 2. List of primers used in this study

Name	Species	Sequence(5'-3')	Notes
RMRP F	human	CAGAGAGTGCCACGTGCATA	
RMRP R	human	CTAGAGGGAGCTGACGGATG	
RPPH1 F	human	GTCACTCCACTCCCATGTCC	
RPPH1 R	human	CGTTCTCTGGGAACTCACCT	
GAPDH F	human	AGCCACATCGCTCAGACAC	
GAPDH R	human	GCCCAATACGACCAAATCC	
18S rRNA F	human	CGAACGTCTGCCCTATCAACTT	
18S rRNA R	human	ACCCGTGGTCACCATGGTA	
MT-RNR2 F	human	AACCACACAACCTACTACCTCA	
MT-RNR2 R	human	AGCCAAAGCTAAGACCCCC	
HEXA F	human	ACGTCCTTTACCCGAACAACT	
HEXA R	human	CGAAAAGCAGGTCACGATAGC	
CCR7 F	human	TGAGGTCACGGACGATTACAT	
CCR7 R	human	GTAGGCCCACGAAACAAATGAT	
MLC1 F	human	CGGGGTTTTCGCTGTACCT	
MLC1 R	human	GGAATCACATTGGCGTTCCTC	
CCR7_WT T	human	CTAGAGCACTCAGCTCTTGGCTCCACTGT	interaction of human *RPPH1* RNA
CCR7_WT B	human	CTAGACAGTGGAGCCAAGAGCTGAGTGCT	
CCR7_Mut T	human	CTAGAGCAAGAAGGCGTTGGCTCTCGAGT	
CCR7_Mut B	human	CTAGACTCGAGAGCCAACGCCTTCTTGCT	
MLC1_WT T	human	CTAGACACCTGCCCCAGGCTCAGGT	
MLC1_WT B	human	CTAGACCTGAGCCTGGGGCAGGTGT	
MLC1_Mut T	human	CTAGACGGTTGCCCCAGGAATGCGT	
MLC1_Mut B	human	CTAGACGCATTCCTGGGGCAACCGT	

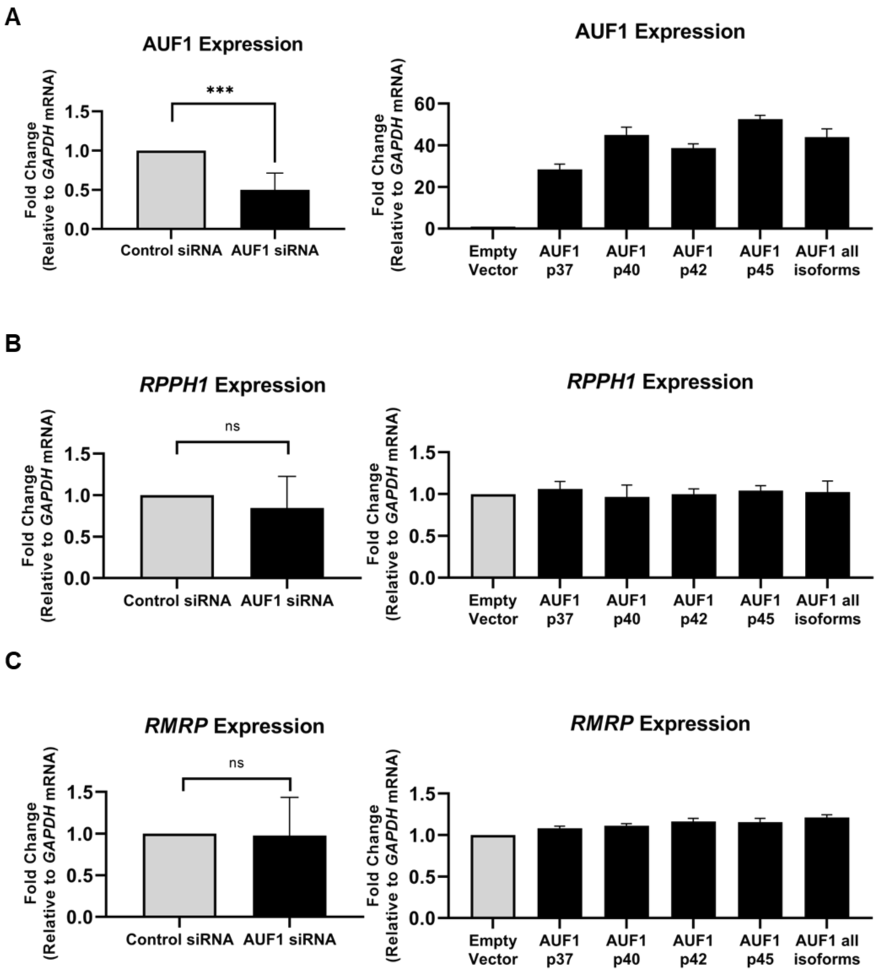

Figure S1. AUF1 does not influence steady state levels of *RPPH1* and *RMPR* RNA.

(**A**) Relative expression of *AUF1* mRNA after siRNA-mediated depletion or overexpression transfected in WI-38 cells (~48h) as measured by whole cell RT-qPCR. (**B**) Relative expression of *RPPH1* RNA after AUF1 depletion or overexpression transfected in WI-38 cells (~48h) as measured by whole cell RT-qPCR. (**C**) Relative expression of *RMRP* RNA after AUF1 depletion or overexpression transfected in WI-38 cells (~48h) as measured by whole cell RT-qPCR. Data in (**A**) to (**C**) is average +/- S.D of independent three experiments. Asterisks denote statistical significance (Student's t test): ns $P > 0.05$, *** $P \leq 0.001$.

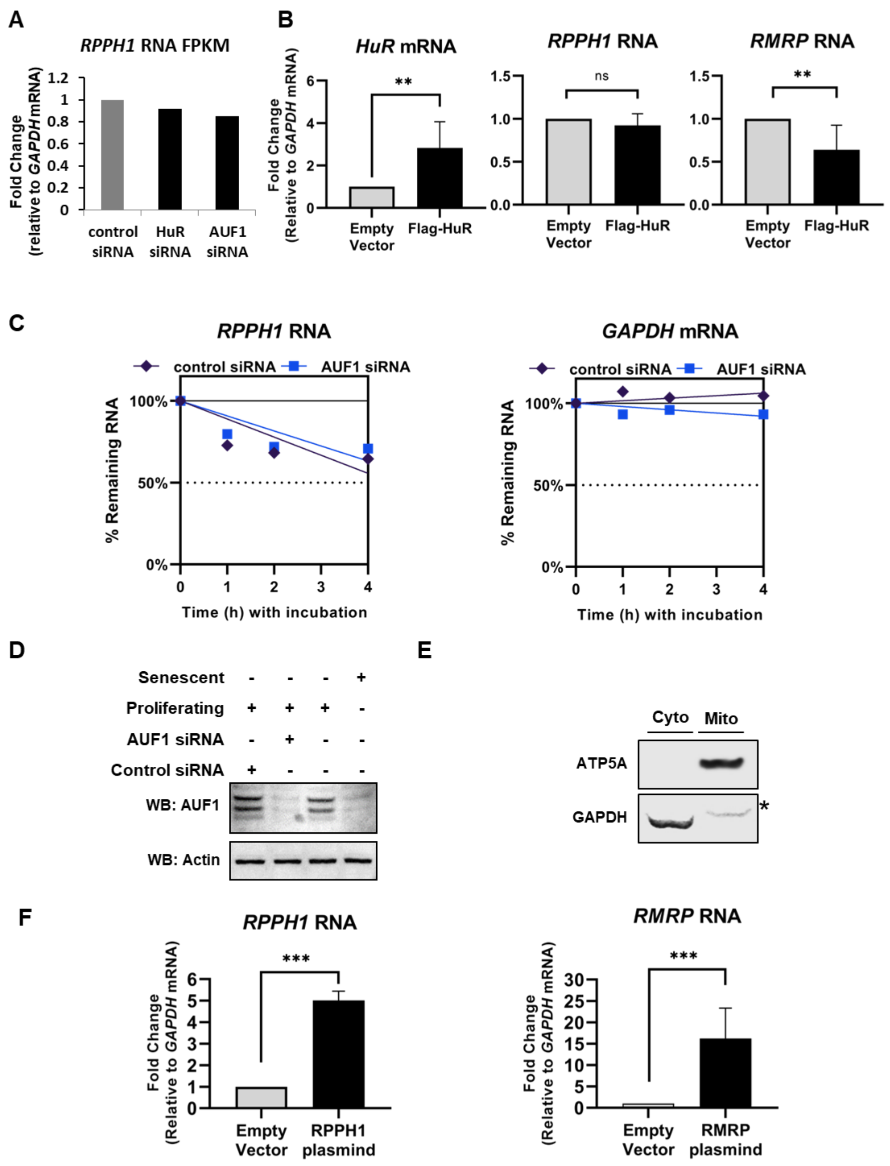

Figure S2. Additional analysis of *RPPH1* RNA, *HuR* mRNA and *RMRP* RNA.

(**A**) Relative FPKM of *RPPH1* RNA in proliferating WI-38 cells after transfection of Control, HuR, or AUF1 siRNA (~48h) normalized to *GAPDH* mRNA. (**B**) Relative expression of *HuR* mRNA, *RPPH1* RNA, and *RMRP* RNA after transfection of Empty Vector or Flag-HuR plasmid as measured by RT-qPCR. Data in (**B**) is average +/- S.D of independent three experiments. Asterisks denote statistical significance (Student's t test): ns $P > 0.05$, ** $P \leq 0.01$. (**C**) Stability of *RPPH1* (left) RNA and *GAPDH* (right) mRNAs after incubation of cell with actinomycin D for the indicated time. (**D**) Relative expression of AUF1 in whole-cell proliferating or senescent WI-38 cells after transfection of Control or AUF1 siRNA. (**E**) Validation of subcellular fractionation by western blot analysis. Asterisk denotes nonspecific band. (**F**) Relative expression of *RPPH1* and *RMRP* RNA overexpression as measured by RT-qPCR. Data in (**F**) is

representative of three independent experiments. Asterisks denote statistical significance (Student's t test): *** $P \leq 0.001$.

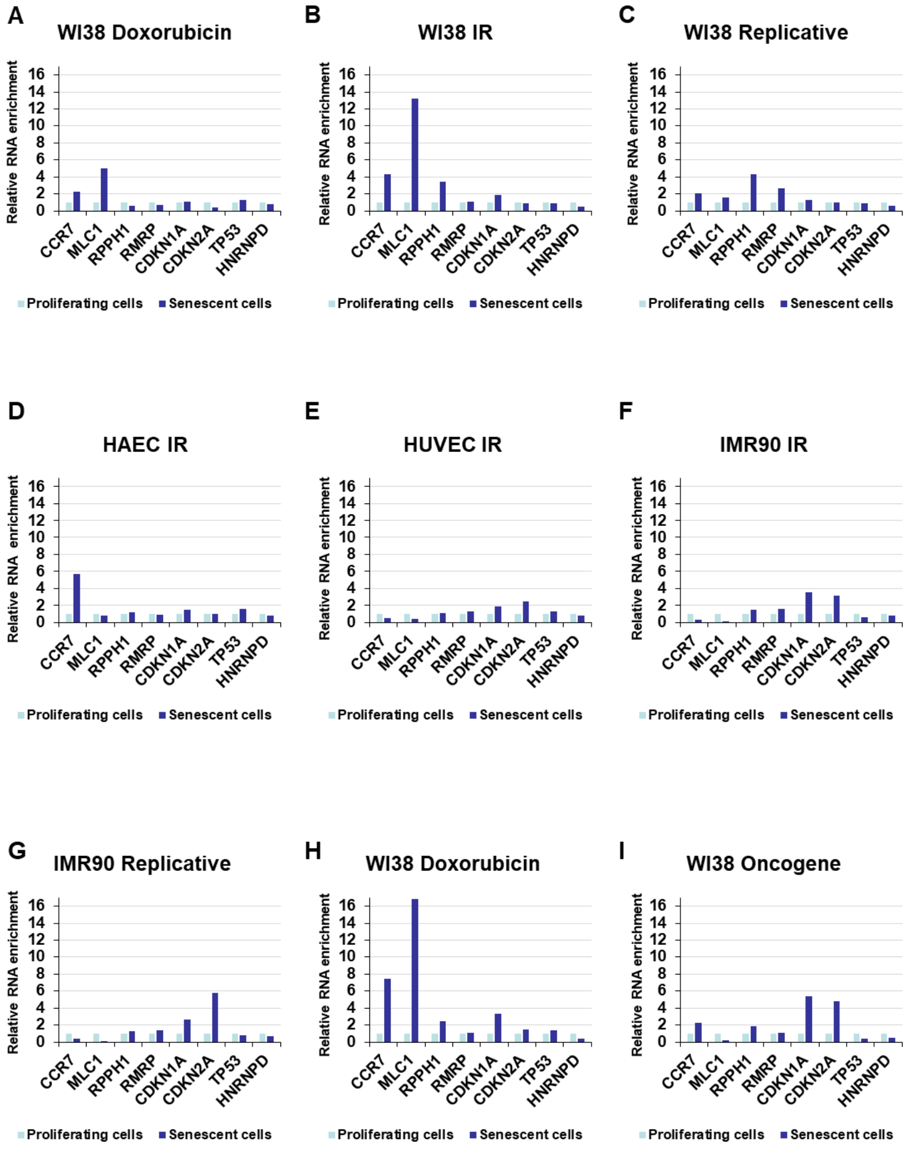

Figure S3. Relative expression of senescence-related RNAs in various cell types and senescence inducers. (**A-I**) Fold changes of FPKM from RNA-seq using total RNAs purified from various cell types before and after senescence induction. *CCR7*, *MLC1*, *p16*, *p21*, *p53* and *AUF1* mRNAs as well as *RPPH1* and *RMRP* RNA were presented.
